# Globin Associated Oxidative Stress

**DOI:** 10.3390/antiox12051077

**Published:** 2023-05-11

**Authors:** Brandon J. Reeder

**Affiliations:** School of Life Sciences, University of Essex, Wivenhoe Park, Colchester CO4 3SQ, UK; reedb@essex.ac.uk

Globins have been studied for their “pseudo-peroxidase” activity for over 70 years, being an ideal model of other kinetically more rapid metalloenzymes. Since those early days of globin redox chemistry research, there has been a realization that globins are much more than oxygen-binding proteins and that they exhibit true redox activities in vivo. Some globins, such as the oxygen-carrying hemoglobin (Hb) of the erythrocyte and myoglobin (Mb) of the myocyte, typically have much of their redox activities suppressed within the confines of the cell. However , under specific conditions, the redox activities of these globins can be manifest, leading to cell and tissue damage through oxidative stress mechanisms including the formation and propagation of free radicals such as the reactive oxygen and nitrogen species (ROS/RNS).

The redox activity of the wider family of globins also has important associations with the mechanisms of cell stress response. Globins may scavenge, generate or sense ROS and RNS as part of their physiological roles. Globins inducing and propagating reactive ROS/RNS under pathological conditions have far-reaching consequences in understanding the cellular response to stress, and in developing strategies to either ameliorate or promote their effects. These mechanisms of oxidative stress and the cellular pathways that they affect continue to be topics of intense interest in both animal and plant diseases.

In this Special Issue of *Antioxidants*, we highlight the most recent developments in the field of globins and similar proteins with respect to their mechanisms of redox chemistry and their contribution or response to oxidative stress. In a review, Alayash examines the evidence and consequences of Hb oxidation reactions and lesions in stored blood and the use of pathogen inactivation technologies [1]. The biochemical changes in stored and irradiated erythrocytes led to time-dependent oxidative challenges, resulting in Hb denaturation and Hb-containing membrane micro-vesicles. The mechanism involves oxidized Hb (via autoxidation or oxidative pathways) binding to band 3 on the erythrocyte membrane, leading to band 3 clustering and driving micro-particulate formation. With an increase in our knowledge of free and intra-erythrocytic Hb oxidative pathways, the redox-dependent damage to aged, stored, inactivated or even stem-cell-derived erythrocytes can now be fully explored and countermeasures designed.

Cellular injuries in atherosclerotic lesions and sickle cell disease (SCD) can be attributed to the ferryl (Fe^IV^) form of Hb, with ferryl sickle Hb (HbS) persisting longer than HbA [1]. Targeting oxidative stress to prevent the damaging effects of HbS in SCD is the topic of the review by Chauhan and Zennadi [2]. Nuclear factor-E2-related factor 2 (Nrf2) can translocate from the cytoplasm to the nucleus to activate the antioxidant response element (ARE) sequences and increases the expression of various cytoprotective antioxidant genes. One such gene is the gamma globin, generating fetal Hb (HbF) that effectively inhibits HbS polymerization and, hence, reduces sickling and oxidative stress. The review also examines the use of various potential treatments to inhibit Kelch-like ECH-associated protein-1 (Keap1) activity, thereby promoting the dissociation of the Nrf2-Keap1 heterodimer and, in turn, activate the ARE. Agents such as dimethyl fumarate, curcumin and tert-butylhydroquinone all exhibit modes of action that lead to the release of Nrf2. While Nrf2-Keap1 signaling is a potential target for SCD treatment to reduce oxidative stress, anemia and painful vasco-occlusive crisis, the exact function of Nrf2 on gamma globin expression remains to be completely defined.

In a study of the antioxidant effects of nitroxyl and nitroxyl anion (HNO/NO^−^) on Hb, Kosmachevskaya et al. show that nitroxyls (from Angeli’s salt) can decrease the levels of glycation by non-enzymatic reactions of Hb through nitrosylation of oxidized Hb and by directly reducing the ferryl form of the globin, decreasing radical propagation and products [3]. Methylglyoxal induced formation of protein carbonyls and advanced glycation end products were decreased by nitroxyl in a dose-dependent way. Additionally, nitroxyl prevents oxidative damage by tert-butyl hydroperoxide. The dual antioxidant and antiglycation effects of the HNO donor are seen as potential pharmacological treatment strategies for decreasing oxidative stress and reversing the stress effect on biomolecules caused by non-enzymatic glycation in carbonyl stress-associated diseases.

Breaking down toxic heme from globins in damaged or senescent cells and intramolecular globins following hemolysis is the primary role of heme oxygenase-1 (HO-1). However, HO-1 can enter the nucleus and, as reported here, is also a DNA-binding protein [4]. Using CHIP-seq, probable DNA binding domain modelling and *Hmox1* deficient cells, Scaffa et al. identified several genes as the most highly connected nodes in the interactome among the HO-1 gene binding targets. The top 10% of these, with the highest *p*-values, all have known connections with HO-1. Specific DNA motifs are identified for HO-1 binding, thereby providing new targets for inhibitors or activators to modulate targeted gene expression and subsequent cellular function.

Cytoglobin (Cygb) increasingly appears to be a multi-functional protein with an apparent but poorly defined role in sensing or protecting against oxidative stress or/and NO regulation. Ukeri et al. explore the in vitro effects of point mutations on the Nitric Oxide Dioxygenase (NOD) and Nitrite Reductase (NiR) activities of Cygb [5]. Here, mutation of the B10 amino acid at the back of the heme pocket decreases the reactivity of Cygb towards NO up to 10^5^-fold, whereas mutation of the distal heme iron amino acid, while creating other effects, increases NiR activity by over 10^3^ fold. This work, together with others, builds a library of mutations to augment specific functions of Cygb under physiological and stress conditions and hence serve as a strategy to develop a more complete understanding of the mechanisms involved in the cytoprotective effects of Cygb in vivo.

One of the roles of Cygb that has seen considerable interest is that of its role in cancer cells and potential resistance to therapy responses. Cygb is consistently downregulated melanomagenesis and its role in melanoma malignancy is investigated De Baker et al. [6]. In the study, the effect of Cygb on the cellular sensitivity towards RAS-selective lethal small molecule (RSL3)-mediated ferroptosis in G361 melanoma cells. Here, Cygb acted as cytoprotectant, ameliorating the outcome of RSL3 treatment by controlling lipid peroxidation and ROS levels. RSL3 treatment increased Nrf2 and downstream HO-1 expression, whereas Cygb levels determined the basal expression of Nrf2 and HO-1. The tumor suppression function of Cygb is supported by transcriptome analysis and is a critical determinant of ferroptosis–pyroptosis therapy response.

Members of the plant-based globin family, the phytoglobins (previously known as non-symbiotic globins), share many structural and functional similarities with their animal counterparts. In many of these globins, conserved cysteines have important roles in the protein’s biochemistry. Christensen et al. examines such a conserved residue in the class 1 phytoglobin from sugar beet (*beta vulgaris*) via mutation (Cys86Ala) [7]. While no significant impact was observed with structure and heme stability, the mutant exhibited significant changes to the autoxidation rate and thermal stability and a decreased, but remarkably stable, peroxidase activity. These effects may be assigned to an altered redox environment of the heme or to a high degree of hexa-coordination (similar to neuroglobin) to account for the observed effects on peroxidase activity the thermostability, respectively. The enhanced autoxidation rate can also be attributed to the conserved cysteine maintaining the redox stability of the iron, transferring electrons from the heme iron to the cysteine, as observed in other globins. This may be direct over the 17.3 Å distance or via pathways involving redox active amino acids such as tyrosine.

Finally, unlike the alpha helical structure of globins, nitrobindins (Nbs) are beta-barrel heme proteins that were discovered less than a decade ago. Nbs are ubiquitous from bacteria to humans and with unclear roles. However, what little information is currently available points to potential roles in RNS regulation, such as nitric oxide trapping and peroxynitrite scavenging, similar to proposed functions of the classical globins. De Simone et al. report on the balance between detoxification of peroxynitrite of ferric Nb from *Danio rerio* and CO_2_ [8]. The pH dependent scavenging of peroxynitrite by Nb leads to nitrite and some nitrite formation. Nb inhibits peroxynitrite-mediated nitration of free L-tyrosine. However, De Simone et al. show that CO_2_, while overwhelming the scavenging activity of Nb for peroxynitrite by facilitating its spontaneous decay, does not impair nitro-L-tyrosine formation. Thus, under normoxic physiological conditions, only circulating erythrocyte Hb is of a concentration to detoxify peroxynitrite. However, it is proposed that under specific environmental conditions, such as those experienced by diving fish, other ferric heme proteins such as Hb may play a relevant physiological role of peroxynitrite scavenging from poorly oxygenated tissue such as the retina.
